# Morphometric brain organization across the human lifespan reveals increased dispersion linked to cognitive performance

**DOI:** 10.1371/journal.pbio.3002647

**Published:** 2024-06-20

**Authors:** Jiao Li, Chao Zhang, Yao Meng, Siqi Yang, Jie Xia, Huafu Chen, Wei Liao

**Affiliations:** 1 The Clinical Hospital of Chengdu Brain Science Institute, School of Life Science and Technology, University of Electronic Science and Technology of China, Chengdu, China; 2 MOE Key Lab for Neuroinformation, High-Field Magnetic Resonance Brain Imaging Key Laboratory of Sichuan Province, University of Electronic Science and Technology of China, Chengdu, China; Massachusetts Institute of Technology, UNITED STATES

## Abstract

The human brain is organized as segregation and integration units and follows complex developmental trajectories throughout life. The cortical manifold provides a new means of studying the brain’s organization in a multidimensional connectivity gradient space. However, how the brain’s morphometric organization changes across the human lifespan remains unclear. Here, leveraging structural magnetic resonance imaging scans from 1,790 healthy individuals aged 8 to 89 years, we investigated age-related global, within- and between-network dispersions to reveal the segregation and integration of brain networks from 3D manifolds based on morphometric similarity network (MSN), combining multiple features conceptualized as a “fingerprint” of an individual’s brain. Developmental trajectories of global dispersion unfolded along patterns of molecular brain organization, such as acetylcholine receptor. Communities were increasingly dispersed with age, reflecting more disassortative morphometric similarity profiles within a community. Increasing within-network dispersion of primary motor and association cortices mediated the influence of age on the cognitive flexibility of executive functions. We also found that the secondary sensory cortices were decreasingly dispersed with the rest of the cortices during aging, possibly indicating a shift of secondary sensory cortices across the human lifespan from an extreme to a more central position in 3D manifolds. Together, our results reveal the age-related segregation and integration of MSN from the perspective of a multidimensional gradient space, providing new insights into lifespan changes in multiple morphometric features of the brain, as well as the influence of such changes on cognitive performance.

## Abstract 2

There is much interest in how the brain’s morphometric organization changes throughout the human lifespan to support cognition. By analyzing structural MRI data from subjects aged 8-89, this study reveals that networks within the brain become more dispersed with age, and that these effects may underlie age-related changes in cognitive flexibility and executive function.

## Introduction

The development of the human brain is an intricate process that underpins cognition [1] and behavior [2,3]. This process is also responsible for brain disorders [4–6]. The brain is neither born mature nor static across the human lifespan [7]. Consequently, regional morphometric changes correspond to chronological human neurodevelopmental changes [8–10]. However, the cerebral cortex comprises parallel, segregated organizations of brain regions central to processing distinct information [11]. The brain connectome indicates that these regional changes complement ongoing maturation of structural networks that involve broad cognitive development or aging processes [12–15].

The developmental trajectory is characterized by significant changes in 2 canonical structural brain connectomes: the white matter fiber tractography [16] and the between-subject morphological/structural covariance network (SCN). The white matter tracts between highly linked hubs are disproportionately influenced by development, such that the frontal-subcortical and frontal-parietal tracts strengthen across late adolescence [15], and the hubs of the brain’s SCN have a long period of adolescence and early adult myelination, with delayed onset of maturity but later onset of decline [17]. However, the white-matter tractography presents challenges, such as estimating the connection strength of long-distance projections, and the SCN generally cannot be used individually [18,19]. These concerns were recently overcome by using a within-subject morphometric similarity network (MSN) [20], which individually captures the interregional correlations of multiple morphometric features [21]. Human MSN modules recapitulate cortical cytoarchitectonic divisions, and regional MSN are related to the co-expression of genes enriched for neuronal terms, axonal connectivity in the macaque, and between-subject variability in human intelligence [21]. A prior investigation using MSN discovered age-related brain quadratic and cubic changes in healthy controls and stimulant use disorders [22]. Therefore, investigating the developmental organization of brain connectomes may provide important insights into typical brain developmental mechanisms and their deviations in neurodevelopmental disorders.

Topographical patterning of the large-scale connectome proceeds gradually in a natural axis of spatial organization through the cortex [23,24]. This gradient axis of the human brain’s architecture is based on various neurobiological features that are important for neurodevelopment [25,26]. Instead of depicting brain boundaries, connectivity gradients characterize each region based on its position along large-scale morphometric gradients. Our previous study of MSN demonstrated that the principal gradient is anchored by motor and sensory cortices at 2 extreme ends and recapitulates fundamental cortical organization properties from gene expression and cyto- and myelo-architecture to evolutionary expansion [27], all of which are evolutionarily rooted. The principle gradient axis, in particular, mirrors a system segregation [23]. The second and third gradients were likewise predicted to illustrate morphometric distinctions of unimodal- and transmodal-related regions. To address age-related changes in multidimensional gradients, a recent study proposed a Euclidean distance to quantify similarities between points within a manifold space, i.e., dispersion [28]. Global, within-network, and between-network dispersion derived from the functional connectivity manifold supports performance in specific cognitive domains across the adult lifespan. Furthermore, within- and between-network dispersions enhance sensitivity to age-related brain changes when compared to other approaches to measuring network changes [28]. To our knowledge, however, no study has explored the age-related changes of dispersion with coordinated morphometric features throughout almost the entire human lifespan.

**Fig 1:**
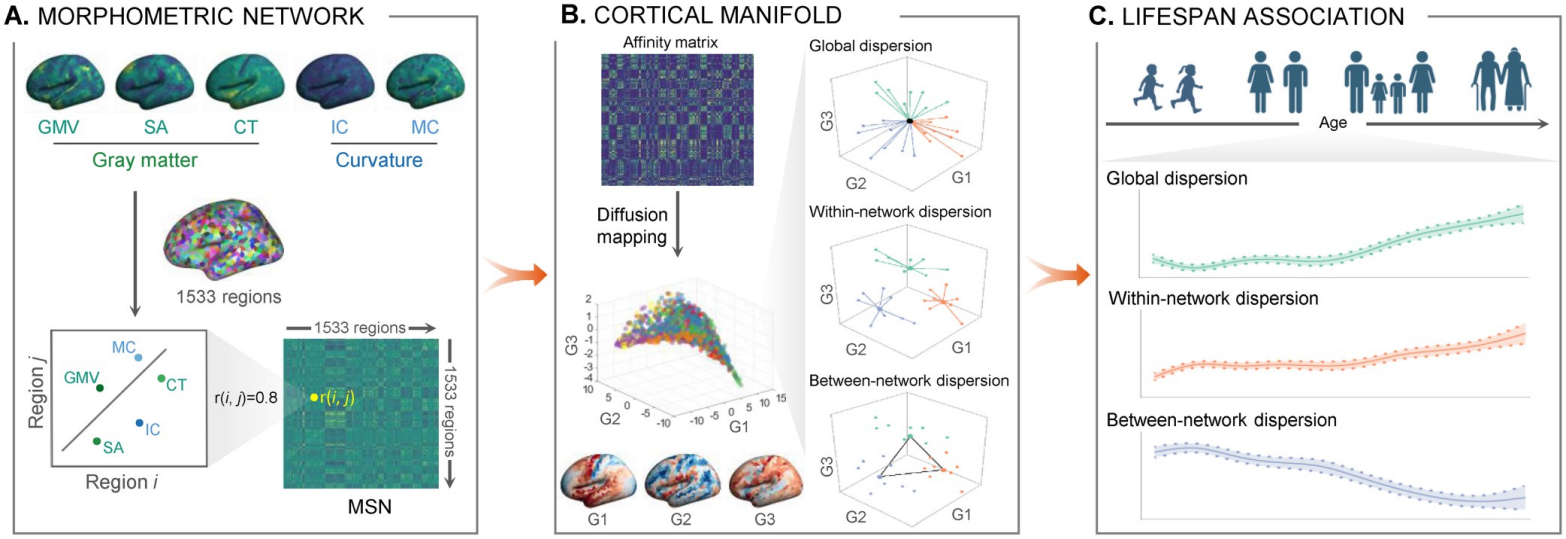
Schematic illustration of this study. **(A)** Construction of an MSN. Using T1w data, we constructed an MSN for each individual using 5 morphometric features, including GMV, SA, CT, intrinsic curvature, and MC. **(B)** Measuring cortical manifold. After obtaining individual MSN, we mapped the connectome into gradient space and calculated the global, within-, and between-network dispersions for each individual from 3D manifold spaces. **(C)** Association lifespan. We used the GAMLSS model to measure age-related changes in brain dispersion across the human lifespan. The cartoons were created with BioRender.com. CT, cortical thickness; GC, Gaussian curvature; GMV, gray matter volume; MC, mean curvature; MSN, morphometric similarity network.

To this end, we used structural neuroimaging in the Human Connectome Project (HCP)-Development (HCP-D), Young Adult (HCP-YA), and Aging (HCP-A) cohorts, which included healthy individuals aged between 5 and 100+ years. Multidimensional morphometric features were constructed for individual MSNs. To extract a global framework from the MSN, we used the dimensionality reduction technique of diffusion map embedding. We initially investigated age-related changes in global dispersion, as well as the link with molecular factors. Then, we assessed whether dispersion of within- and between-networks changed as we age. Finally, we examined how the global, within-, and between-network dispersions were linked to cognitive domains [29,30]. The analysis schematic is shown in Fig 1.

## Results

In HCP-D (*n* = 652), 13 participants under the age of 8 years were excluded because of insufficient data available for 5 to 7 year olds. For HCP-YA (*n* = 1,206), 93 participants without T1-weighted images (T1w) and 668 participants with siblings in the same family were excluded to reduce bias. For HCP-A (*n* = 725), 13 participants over the age of 89 were excluded because of the data non-availability. A flowchart of the study participants is shown in S1 Fig. Finally, 1,790 participants (mean age: 35.67 years; age range: 8 to 89 years; 974 females) were included (S2 Fig).

In accordance with our prior work, 5 morphometric features were extracted from 1,533 cortical regions and used to construct individual MSNs [27]. We also constructed the MSN matrix using 7 features (2 more features from diffusion-weighted imaging) to test the robustness of the MSN (S1 Table). We found a comparable MSN pattern derived from 5 and 7 features across subjects (*r*[mean ± standard deviation] = 0.70 ± 0.03; S3 Fig). The top 3 embedding components (gradients) were then identified by computing cortical connectivity gradients using a diffusion map embedding technique. The gradients described principal spatial axes along which morphometric similarities varied across cortices. Regions that resembled each other with respect to morphometric features occupied similar positions along the gradient. Individualized MSN gradients were aligned to a template generated by averaging all MSNs using a ComBat harmonization due to variance in magnetic resonance imaging (MRI) collecting procedures [31]. Morphometric communities were compactly localized in the group-average 3D gradient space (Fig 1). The principal gradient (G1) drove the insular cortices to the extreme and segregated locations. The second gradient (G2) likewise drove the association cortices to extreme locations. The secondary sensory class occupied one side of the third gradient (G3).

### Age-related differences in global dispersion

**Fig 2:**
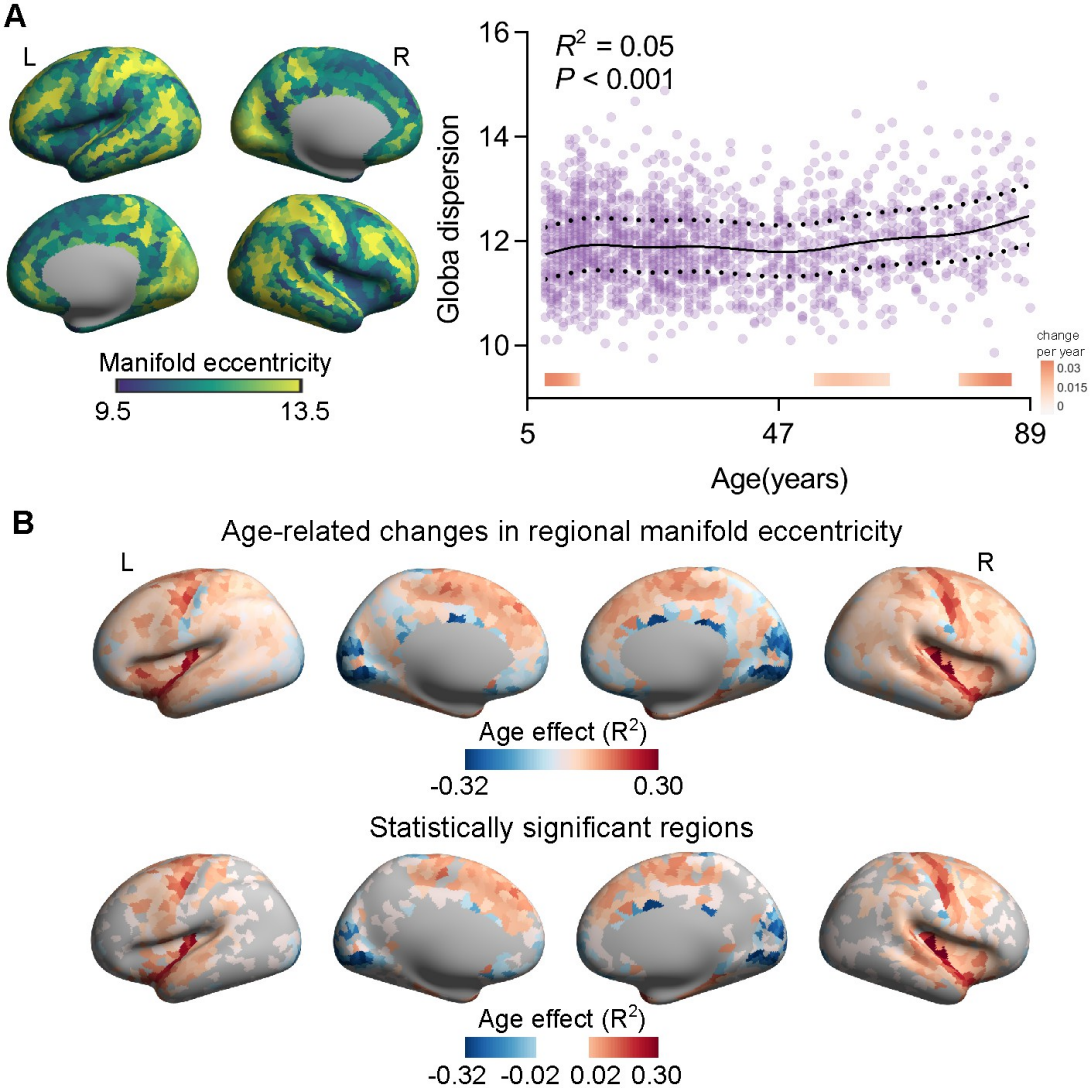
Global dispersion of the MSN connectome. **(A)** Distribution of average manifold eccentricities and age-related changes in global dispersions across the human lifespan. The dotted lines represent 25% and 75% centiles. The filled bar above the x-axis depicts the derivative of the GAMLSS and corresponds to developmental windows of significant global dispersion. The red bar color represents significant global dispersion increases, and the blue bar color represents significant global dispersion decreases. **(B)** Regional manifold eccentricities measured by Euclidean distance between the center and each region. The age effects (R^2^ values) were the age-related regional manifold eccentricities across the human lifespan. Statistically significant regions were corrected by FDR. The data underlying this figure can be found in S1 Data. FDR, false discovery rate; MSN, morphometric similarity network.

We first investigated whether global dispersion, defined as the sum of squared Euclidean distances of all regions to the global centroid in the 3D cortical manifold, changed with age. A small/large global dispersion value indicated that the overall morphometric similarity profiles across all regions diverged by a low/high amount along 3D gradients. We used a generalized additive model for location, scale, and shape (GALSS), a robust method for modeling nonlinear growth trajectories [7], to construct brain charts for the human lifespan. After controlling sex and estimated total intracranial volume (eTIV), we found that the global dispersion increased with age (*R*^2^ = 0.05, *P* = 6e-6; Fig 2A), and the development trajectory was similar to the MSN matrix derived from 7 features (S4 Fig). To determine the developmental windows of significant morphometric network integration, we measured the first derivative of the age smooth term, which indicates the change in global dispersion at a given age [32,33]. Age-fit derivatives revealed distinct timings of developmental changes in different dispersions of MSN. The global dispersion increased most significantly during childhood to adolescence and late adulthood (Fig 2A).

Furthermore, we segmented participants into 4 age windows (late childhood to adolescence: 8 to 19 years, young adulthood: 20 to 39 years, middle adulthood: 40 to 59 years, and late adulthood: 60 to 89 years) based on a previous study [7], to assess age-dispersion relationships for each age group. Similarly, we found an increase in global dispersions during adolescence and late adulthood and a relatively stable tendency during young and middle adulthood (S5 Fig). Given the importance of executive functions (EFs) in human development [34], we conducted a mediation analysis to explore whether the influence of age on EFs might be explained by changes in global dispersions. We utilized 2 core EFs: cognitive flexibility and inhibition control. For significance testing, bootstrapping was used. We found that the increase in global dispersion was associated with age (path a: β = 0.003, *P* = 0.002), and lower cognitive flexibility in EF was related to age (path c: β = −0.093, *P* < 0.001). There was no association between global dispersion and cognitive flexibility in EFs (path b: β = −0.211, *P* = 0.563) after adjusting for age. Mediation analysis, however, revealed that global dispersion was not a significant mediator (indirect effect = −0.001, *P* = 0.570). In terms of inhibition control of EFs, we found that an increase in global dispersion was associated with age (path a: β = 0.003, *P* = 0.002), and lower cognitive flexibility of EFs was related to age (path c: β = −0.102, *P* < 0.001). There was no association between global dispersion and cognitive flexibility in EF (path b: β = 0.093, *P* = 0.764) after age adjustment. However, mediation analysis revealed that global dispersion was not a significant mediator (indirect effect <0.001, *P* = 0.765).

Furthermore, we found that during human aging, cortical regions exhibited unique patterns of increased (i.e., expansion) and decreased (i.e., contraction) global dispersions (Fig 2B, upper panel). To identify which regions showed significant changes in global dispersions across the human lifespan, we corrected for multiple comparisons using false discovery rate (FDR) correction (*q* < 0.05). We found that regions within the secondary sensory cortices showed substantial declines across the cortical cortex, indicating that these regions were morphometrically segregated from the rest of the brain during aging (Fig 2B, lower panel). However, dorsal prefrontal and insular cortices expanded significantly and increased with age, presumably indicating that these regions with increased morphometrically segregated from the other regions during aging.

### Associations with neurotransmitter receptors and gene expression patterns

**Fig 3:**
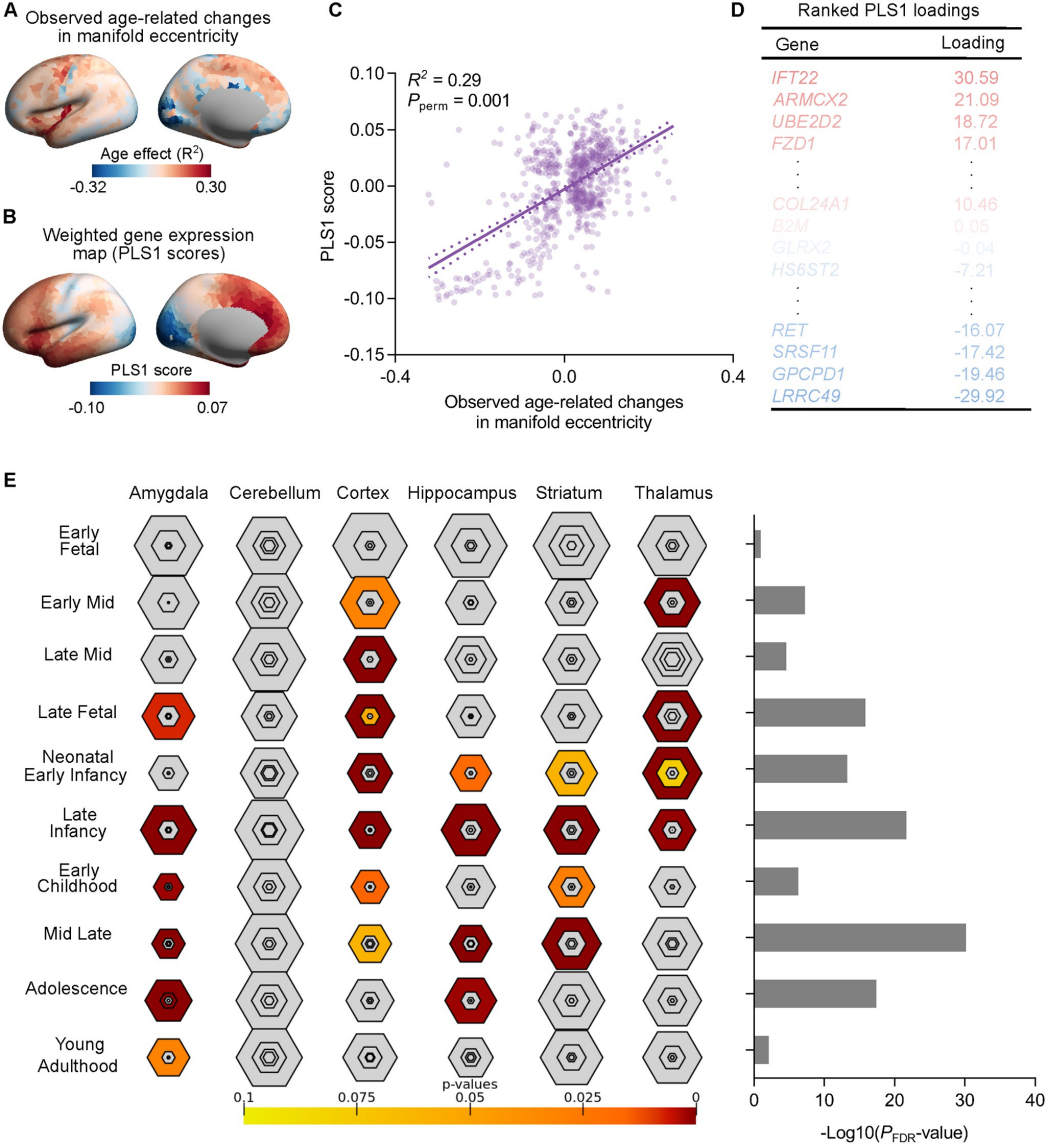
Transcriptomic analysis. **(A)** Age-related changes in regional manifold eccentricity in the left hemisphere (unthresholded). **(B)** A weighted regional gene expression map of the first component of PLS (PLS1) scores in the left hemisphere (unthresholded). **(C)** A scatterplot of regional PLS1 scores and age-related changes in regional manifold eccentricity (*R*^2^ = 0.29, *P*_perm_ = 0.001). **(D)** Ranked PLS1 loadings. **(E)** The top 1,000 genes (*P*_FDR_ < 0.05) were used in development analysis, showing strong associations with the cortex and amygdala during early childhood to adolescence. The data underlying this figure can be found in S1 Data. FDR, false discovery rate; PLS, partial least squares.

We next quantified the contributions of molecular factors to global dispersions. The spatiotemporal gene expression trajectories reveal human cortical developmental hierarchy [35]. We thus used transcriptomic and developmental enrichment analyses to contextualize the age-related global dispersions to patterns of postmortem gene expressions from the Allen Human Brain Atlas (AHBA), which is a whole-brain transcriptomic dataset. We used partial least squares (PLS) regression analyses to determine the relationships between age-related global dispersions (Fig 3A) and gene expressions (7,645 genes). The first component of PLS (PLS1) explained 29% of the variance (*P*_spin_ = 0.005; Fig 3B). The PLS1 weighted gene expression map spatially correlated with the age-related global dispersion map (*R*^2^ = 0.29, *P*_perm_ = 0.001; Fig 3C). We ranked the normalized weights of PLS1 based on multiple univariate Z tests. Among the list of most strongly associated genes (*P*_FDR_ < 0.05; Fig 3D), we selected the top 1,000 genes and performed developmental gene set enrichment analysis using the cell type-specific expression analysis (CSEA) tool, which compared the selected gene list with development enrichment profiles. This analysis highlights developmental time windows across macroscopic brain regions where genes are strongly expressed. We found marked expression of genes enriched from late infancy onward in the amygdala, striatum, and hippocampus (Fig 3E). Genes were also enriched for expression in the thalamus during transition from early fetal life to late infancy. However, the identified genes were not expressed in the cerebellum.

**Fig 4:**
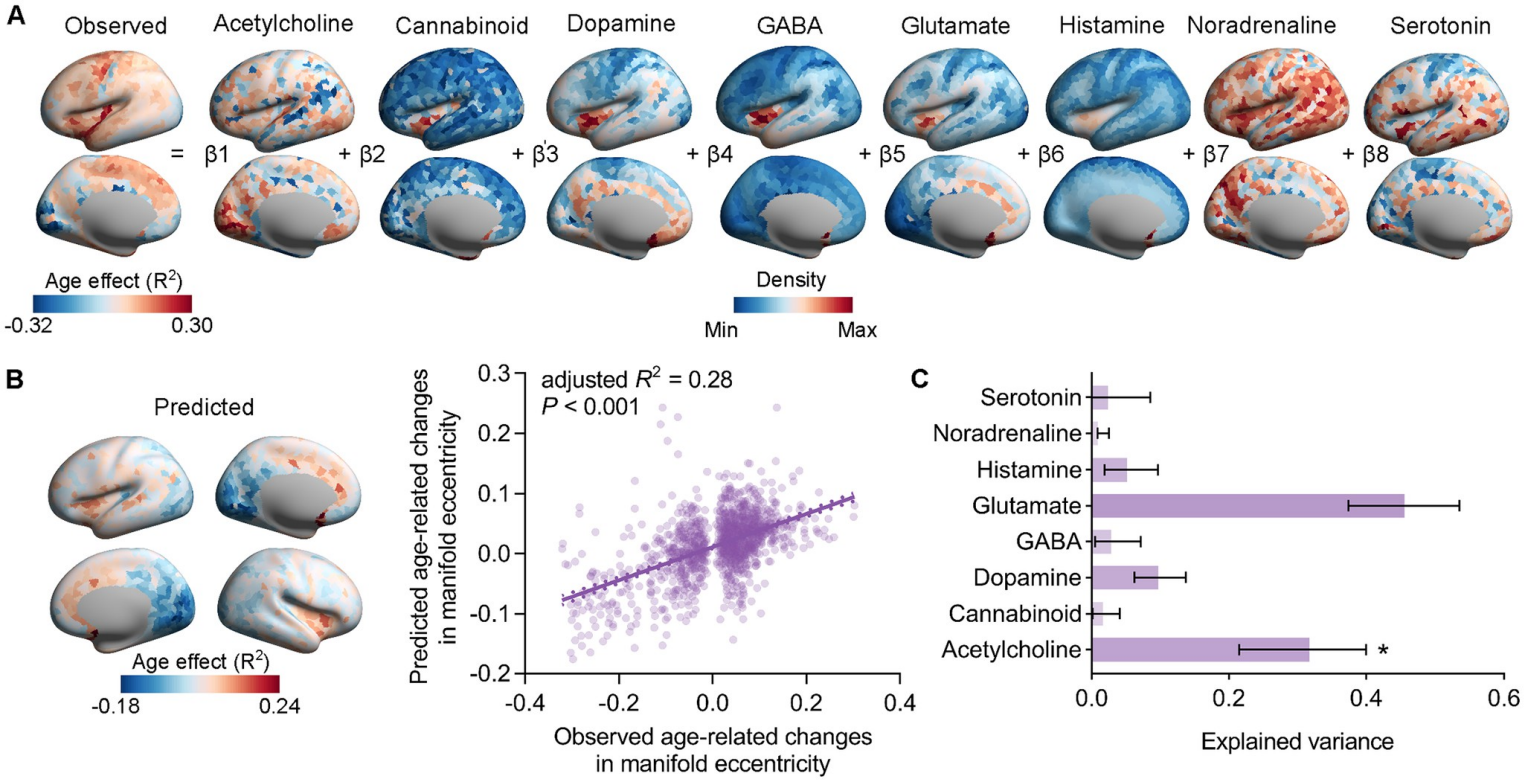
Contributions of neurotransmitter receptors to age-related changes in regional manifold eccentricity. **(A)** A multiple linear regression model was used to determine the relationships between neurotransmitter receptors and age-related changes in regional manifold eccentricity. **(B)** The predicted age-related changes in regional manifold eccentricity and a plot of the predicted versus observed *R*^2^-values (adjusted *R*^2^ = 0.28, *P* < 0.001). **(C)** The relative importance of each neurotransmitter receptor contributed to the multiple linear regression model. Error bars denote 95% bootstrap CIs. Asterisks indicate a statistically significant contributor. The data underlying this figure can be found in S1 Data. CI, confidence interval.

Neurotransmitter receptors drive synaptic plasticity, modify neural states, and ultimately form network-wide communications [36]. However, it is uncertain how the patterning of different neurotransmitter receptors relates to global dispersion across the human lifespan. We used 8 distinct neurotransmitter systems using positron emission tomography (PET) data [36], including serotonin, dopamine, histamine, acetylcholine, cannabinoid, glutamate, GABA, and noradrenaline. After assigning the receptor distribution maps to DK-1,533 brain regions, we used a multiple linear regression model combining all 8 neurotransmitter systems using the *relaimpo* package in R (Fig 4A). The model explained 28% of the variance in the global dispersion across the human lifespan (*F*_(8, 1524)_ = 72.8, *P*_spin_ = 0.0008; Fig 4B). Furthermore, we found that acetylcholine (*P*_spin_ = 0.0032, FDR-correction) receptor can predict global dispersions, and dopamine (*P*_spin_ = 0.07, FDR-correction) and glutamate (*P*_spin_ = 0.07, FDR-correction) receptors marginally predicted global dispersion (S2 Table). Notably, in our model, the glutamate receptor exhibited the largest dominance in global dispersions (Fig 4C).

### Within- and between-networks dispersion changes across the human lifespan

**Fig 5:**
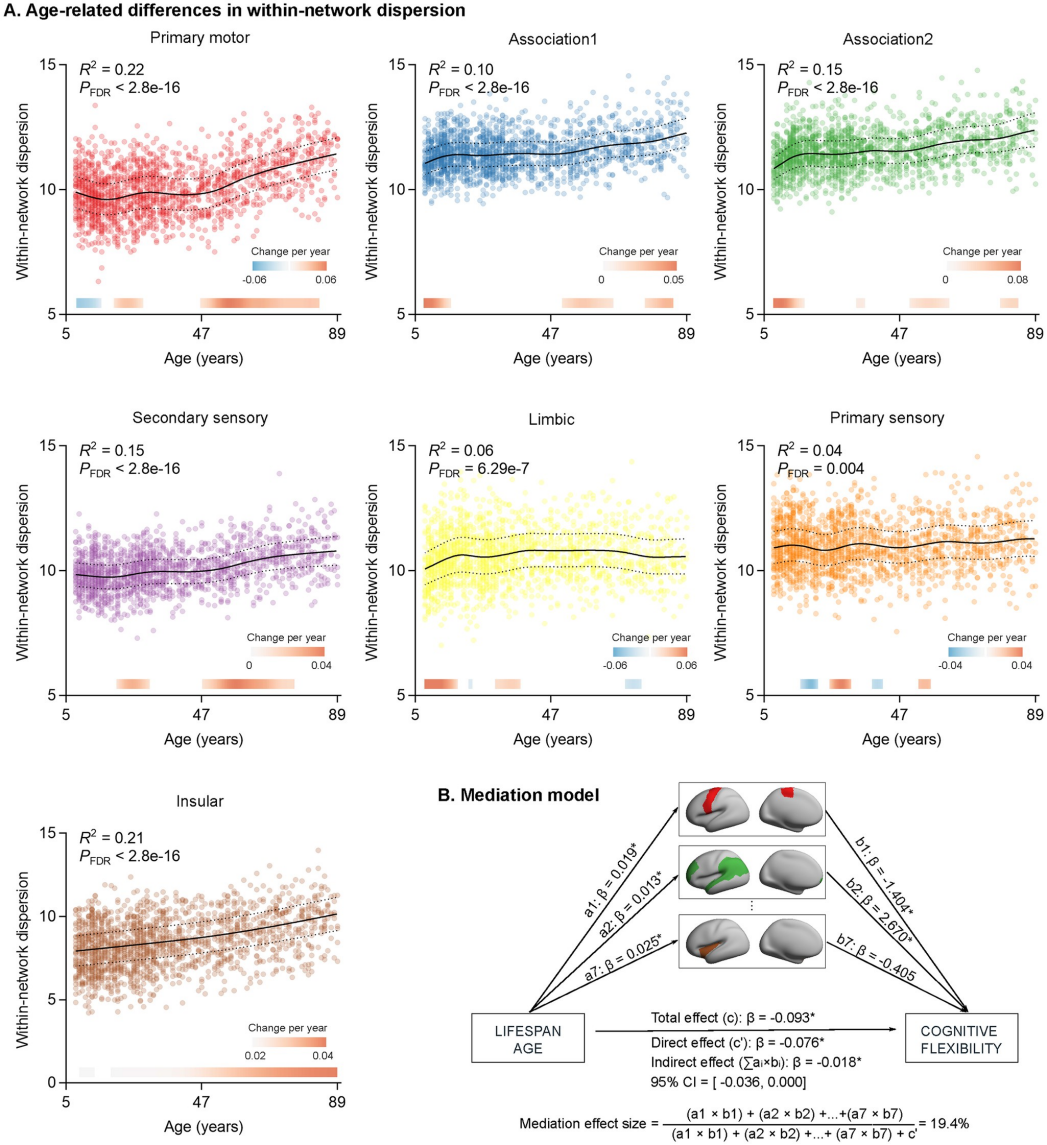
Age-related changes in within-network dispersions. **(A)** The associations were measured in 7 cytoarchitecture classes, whose dispersions increased with age. The dotted lines represent 25% and 75% centiles. The filled bar above the x-axis depicts the derivative of the GAMLSS and corresponds to developmental windows of significant within-network dispersion. The red bar color represents significant within-network dispersion increases, and the blue bar color represents significant within-network dispersion decreases. **(B)** Within-network dispersion mediated the influence of age on the cognitive flexibility of EFs. The data underlying this figure can be found in S1 Data. EF, executive function.

Age-related differences for within- and between-network dispersions were estimated for the von Economo cytoarchitectonic parcellation with 7 networks. We used GAMLSS to depict the relationships of age-related differences, similar to the fitting curve of global dispersions, showing that all networks exhibited increased overall within-network dispersion with increasing age (S3 Table). Most networks followed nonlinear within-network morphometric similarity patterns (Fig 5A). After identifying the developmental windows of significant within-network dispersion, we found that the association and limbic cortices increased rapidly during late childhood to adolescence, and within-network dispersion of primary motor and secondary sensory cortices increased rapidly during middle adulthood.

Furthermore, we evaluated the developmental trajectories in 4 age windows. The primary motor and secondary sensory classes showed decreases within-network dispersion during late childhood to adolescence and increases from young adulthood to late adulthood (S6 Fig). The dispersion of the primary motor class increased rapidly during late adulthood, and the secondary sensory class increased rapidly during young adulthood (S4 Table). The association 1 and association 2 classes showed a steep ascent of within-network dispersions from late childhood to adolescence, with a slower ascent thereafter. The limbic class also showed a steep rise of within-network dispersion from late childhood to adolescence, but a slower descent during late adulthood. The primary sensory and limbic classes exhibited increased within-network dispersions across the whole age and increased rapidly during young adulthood. To further validate these results, we used the Yeo-7 functional atlas. Similar to the von Economo atlas, we found that most functional networks were significantly increased with age, especially within-network dispersions of frontoparietal, default mode, and dorsal attention networks during adolescence. However, the within-network dispersions of a limbic class increased during adolescence and thereafter decreased from young adulthood to late adulthood (S7 Fig).

Furthermore, we tested whether within-network dispersions mediated the effects of age on EF using multilevel mediation analysis. The joint effects of age on within-network dispersions (path a) and within-network dispersions on EFs (path b), as well as the total (path c) age effect on EFs, were examined. In addition, bootstrap analyses were performed to assess the statistical significance of the mediation analysis, for which a 95% confidence interval (CI) without zero was equivalent to a significance level of 0.05. After accounting for within-network dispersions, the effect of age on cognitive flexibility of EFs was weakened (path c’: *β* = −0.076, *P* < 0.001, from path c: *β* = −0.093, *P* < 0.001). Age-related differences in cognitive flexibility of EFs were mediated by the primary motor class (path a: β = 0.019, *P*_FDR_ < 0.001; path b: β = −1.404, *P*_FDR_ < 0.001; indirect effect = −0.027, 95% CI = [−0.041, −0.013], *P*_FDR_ < 0.001) and association cortices (path a: β = 0.013, *P*_FDR_ < 0.001; path b: β = 2.670, *P*_FDR_ = 0.007; indirect effect = 0.034, 95% CI = [0.013, 0.058], *P*_FDR_ = 0.008) (S5 Table). Computing the effect size yielded a mediation effect of 19.4% variance (Fig 5B), corresponding to a medium to large effect size. In addition, we found that the 2 classes jointly mediated the relationships between age and the cognitive flexibility of EFs (indirect effect = 0.032, 95% CI = [0.001, 0.062], *P* = 0.04; direct effect = 0.519, 95% CI = [0.448, 0.590], *P* < 0.001) during late childhood to adolescence. However, when within-network dispersion was considered, the age effect on inhibition control of EFs remained relatively stable (path c’: β = −0.16, *P* < 0.001, from path c: β = −0.162, *P* < 0.001), and within-network dispersion did not mediate the relationships between age and inhibition control of EFs (indirect effect = −0.002, *P* = 0.799).

**Fig 6:**
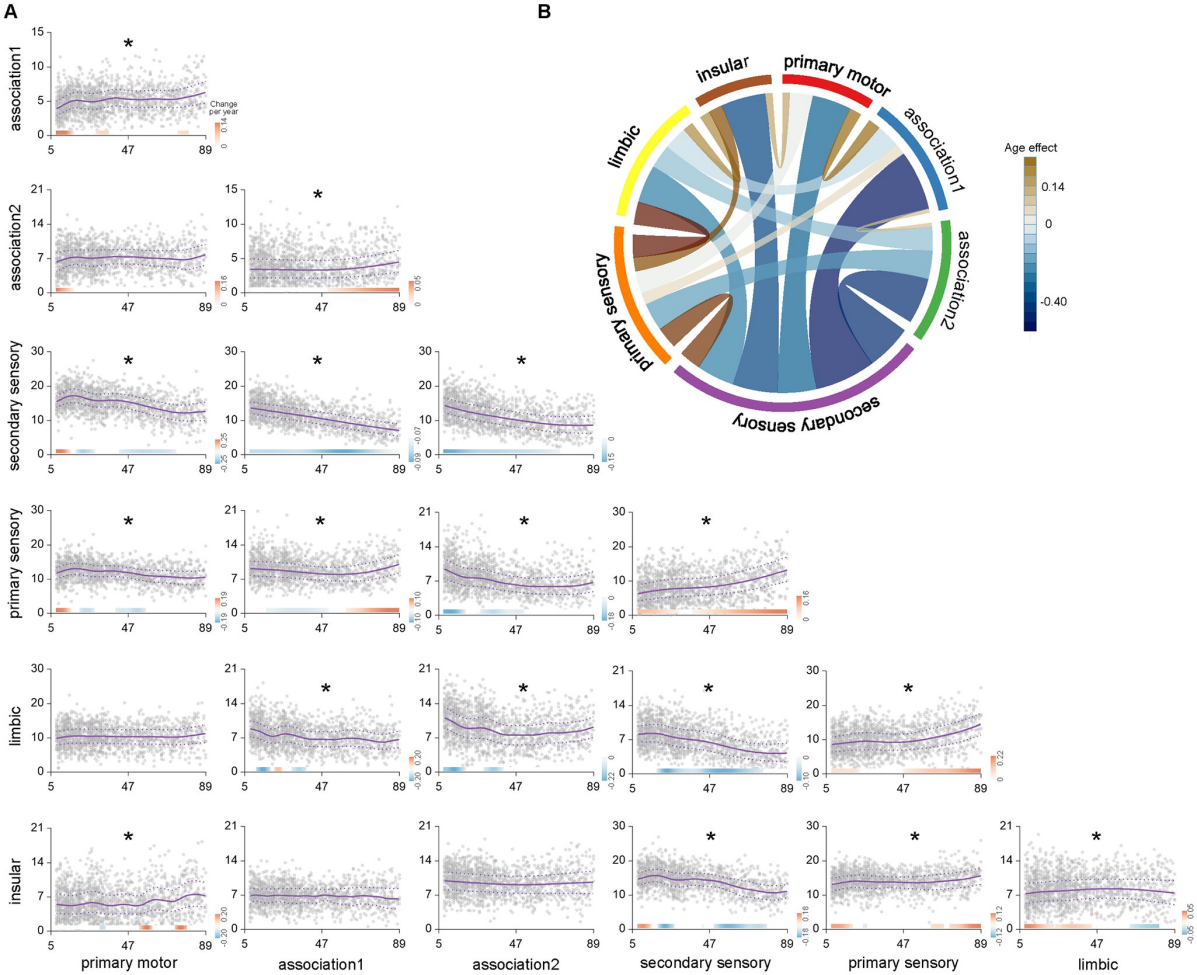
Age-related changes in between-network dispersions. **(A)** Nonlinear between cortical network age-related differences in dispersions with a statistically significant nonlinear relationship with age accounting for sex and eTIV as covariates. The dotted lines represent 25% and 75% centiles. The filled bar above the x-axis depicts the derivative of the GAMLSS and corresponds to developmental windows of significant between-network dispersion. The red bar color represents significant between-network dispersion increases, and the blue bar color represents significant between-network dispersion decreases. **(B)** Distribution of the R^2^-values of between-network dispersion across the human lifespan. Network borders are scaled according to the size of the total effect from communities. The data underlying this figure can be found in S1 Data. eTIV, estimated total intracranial volume.

Nonlinear between-network dispersion (the distance between the centroids of each network) usually occurred between primary and secondary sensory classes, as well as other networks (Fig 6 and S6 Table). Between-network dispersions in association-secondary sensory and association-limbic cortices exhibited curvilinear decreases with age. Except for the dispersion between association 1 and secondary sensory classes, which showed the most descent in late adulthood, the rapid descent stage of other between-network dispersions occurred during late childhood to adolescence (S8 Fig and S7 Table). The dispersion between association cortices and primary sensory classes decreased from late childhood to middle adulthood, while they increased during late adulthood. The between-network dispersions of primary motor-association cortices and primary motor-limbic significantly increased during late childhood to adolescence, and the dispersions of primary motor-secondary sensory and primary motor-primary sensory significantly increased until adolescence, then decreased thereafter. Similarly, the secondary sensory-limbic and secondary sensory-insular exhibited increases in between-network dispersions during adolescence, and the decreased thereafter. The secondary sensory-primary sensory exhibited an increased trend and increase in accelerating trend during late adulthood. Similar to the von Economo atlas, most between-network dispersions defined by Yeo-7 functional atlas decreased with age, especially between the visual network and other networks (S9 Fig). However, between-network dispersion was not the mediator between age and the cognitive flexibility of EFs (indirect effect = 0.017, *P* = 0.232) and inhibition control of EFs (indirect effect = 0.018, *P* = 0.166).

## Discussion

Using the cortical manifold of the MSN, we evaluated lifespan age-related differences in 3D gradient dispersions. Global dispersion gradually increased with increasing age. The manifold eccentricity of visual cortices—the distance between visual cortices and global centroids—contracted as age increased, whereas manifold eccentricities of prefrontal cortices expanded during aging. Molecular neurotransmitter systems have recognized that spatial distribution of multiple neurobiological systems, including glutamate, dopamine, and acetylcholine neurotransmitters, recapitulated the age-related differences in global dispersions. Decoding the global dispersions with postmortem gene expression maps implicated genes enriched in middle/late childhood and adolescence, again identifying to both cortical and subcortical targets. In addition, we found that all within-network dispersions gradually increased with age. With the exception of primary sensory cortices, secondary sensory cortices almost uniformly moved closer toward the center of all other networks. Finally, within-network dispersions, including primary motor, association, and insular cortices could mediate the effect of age on the cognitive flexibility of EFs. Taken together, our results showed the importance of MSN integration for EF from a multidimensional perspective.

By leveraging advanced manifold learning, we could depict macroscale MSN connectome organization along principal axes. This technique provided a valuable perspective to bridge low-dimensional representations of cortical organizations and human cognition in a data-driven and spatially unconstrained manner [14,23]. The MSN connectome shared topological properties with structural and functional networks, such as community structure, degree distribution, and rich club [21]. Furthermore, MSN abnormalities have been reported to be involved in numerous brain disorders [37–40]. However, our prior study reported that the principal MSN gradient was anchored by motor and sensory cortices at 2 extreme ends [27]. Notably, the MSN gradient fundamentally recapitulated cortical organization, ranging from gene expression and cyto- and myeloarchitecture to evolutionary expansion, which showed similar patterns to human development [41]. Because gradient dispersion is sensitive enough to detect changes in brain connectomes during individual progression [28], we were able to show age-related changes in cortical dispersions of MSN connectomes, which added our understanding of morphometric segregation across the human lifespan using multiple modality features in multidimensional space. Age-related differences in global dispersions therefore increased across the human lifespan. Increased dispersions captured increased differentiation of overall MSN profiles across regions in the 3D cortical spaces of the MSN connectome. Global dispersion reflects network segregation at the whole-brain level. Consistent with an earlier study [42], we found that global dispersion increased during late childhood to adolescence, indicating an enhanced discrete distribution of brain regions in gradient space. Previous functional findings reported that in older adults, the visual cortices showed signs of hyperactivity combined with decreased activation in control and default mode networks [28,43]. Notably, after measuring eccentricity for each region, we found that age-related changes were primarily characterized by manifold contraction (i.e., decreased dispersions) of secondary sensory cortices, as well as expansion (i.e., increased dispersions) of transmodal areas of the prefrontal and insular cortices, which might fit with the prior findings of increasing differentiation of higher-order association cortices from the rest of the brain, based on structural connectomes [14]. Thus, at 3D manifolds, we observed increased global dispersions across the human lifespan.

Brain development encompasses numerous processes, such as synaptogenesis, migration, and synaptic plasticity. These activities are regulated by neurotransmitters, such as glutamate receptors. In the present study, glutamate receptors were primarily responsible for the age-related changes in manifold eccentricity. Glutamate is the major excitatory neurotransmitter in adults, mediating neuronal communication in the nervous system during human development [44]. Besides its unique role, the glutamate receptor is co-localized in many brain regions [45], and its balanced interaction with GABA receptors is crucial for normal brain development [46]. Thus, deficit or dysregulation of glutamate receptors may affect an individual’s subsequent development and normal functioning, leading to various neurodevelopmental and psychiatric disorders [47–49]. In addition, we found that dopamine and acetylcholine receptors significantly contributed to manifold eccentricity changes across all aspects of human development. Acetylcholine, as a neuromodulator, is essential in the developing and mature brain [50]. A recent study reported that ionotropic nicotinic receptors, where acetylcholine signals through this class of receptors, contributed to the maturation of glutamatergic synapses, indicating the critical role of acetylcholine signaling in synaptic development [51]. Dopamine plays an important role in cognition, learning, and memory. Dysfunctions of the frontal cortical dopamine system have been implicated in several developmental neuropsychiatric disorders [52]. Elucidating the relationships between age-related differences and neurotransmitters in the brain might therefore help us to better understand the precise function of neurotransmitter receptors during development and mature brain processing.

By associating macroscopic changes in manifold eccentricity with microscopic gene expressions provided by the AHBA, we identified gene sets expressed in cortical regions, amygdala, and hippocampus during middle and late childhood and adolescence. Brain development and function depend on the precise regulation of gene expressions [53]. Spatial-temporal waves of gene expression changes are involved in different brain regions and human developmental time windows [53,54]. A previous study reported that coupled brain networks and molecular changes may ultimately affect cortical and subcortical circuit properties, including the balance of excitation (glutamatergic) and inhibition (GABAergic) (E/I) [14]. During adolescence, E/I balance shifts the dominant inhibitory bias in early developmental stages toward stronger excitatory drive in later stages, suggesting that these may be responsible for the maturation of working memory and executive control functions [55–57]. A transient E/I imbalance during early development generally underlies the development of several neurological and psychiatric disorders, such as autism, schizophrenia, and attention-deficit hyperactivity disorder. Despite these findings being associative and based on a separate dataset, they support our results that brain network changes across the human lifespan implicate micro- and macroscale factors in both cortical and subcortical networks.

Cortical within-network dispersions increased nonlinearly with age, possibly reflecting more complicated age-related stages of morphometric refinements. The strongest increased within-network dispersions were observed in the insular and primary motor cortices, and in the association and secondary sensory cortices. Increased dispersion captures decreased similarity within specific networks across multidimensional spaces of the MSN connectome. This finding aligns well with prior reports of broadly weakened within-network connectivities across the human lifespan [58]. Specifically, during late childhood to adolescence, increased within-network dispersions in association cortices and decreased dispersions within primary motor and secondary sensory classes were consistent with previous studies [26,59], suggesting that multidimensional within-network dispersion development occurred along the sensorimotor-association cortical axis from late childhood to adolescent. In addition, primary motor and secondary sensory classes exhibited cubic developmental trends from adolescence and middle adulthood, and then slowly increased during late adulthood, whereas association cortices increased rapidly during adolescence, with a slower increase thereafter. The differences suggested the existence of network-specific dispersion development trajectories with age [28]. Combining these results with previous findings that EFs relied on complex and continuous interactions within the human brain connectome and declined with age [34], we speculated that age-related changes in within-network dispersion were closely associated with the age-accompanied declines in EFs. We found that motor, insular, and association cortices mediated negative associations of age with the cognitive flexibility of EFs, suggesting a central role for attention and frontoparietal control systems in the maturation of the normal EFs [34]. In addition, consistent with previous findings of increased brain connectivities during aging [58], we found a nonlinear decrease in between-network dispersions, which was mainly focused on secondary sensory cortices. Similar to the global dispersion findings, the secondary sensory cortices became more like the rest of the cortices, moving closer to the center of other networks across the human lifespan [28]. However, with increasing age, we found increased morphometric dispersions between several networks, particularly between primary sensory and secondary sensory, and limbic, association cortices, as well as between association cortices. This finding was consistent with a prior report that increased structural differentiation between default mode and attention, frontoparietal regions, as well as between sensory and limbic, ventral attention, and visual networks [13], likely reflected ongoing morphometric segregations of primary and heteromodal systems. Thus, the within- and between-network dispersions might serve as a biomarker for aging. Together, these results further confirmed our conjecture that age-related changes in network dispersion may represent healthy EFs change across the human lifespan.

Some methodological considerations should be noted. First, the present findings were based on a cross-sectional cohort, but we could not directly investigate individualized dispersion changes over time. Thus, our discussion focused on the effects of age and its correlations. Future studies should use longitudinal cohorts to confirm whether the gradient dispersion can detect changes in morphometric reorganization during individual progressions. The second limitation was that participants younger than 8 years and older than 89 years were excluded due to the limited number of participants in these age groups. Future studies should include a greater number of participants, to investigate age-related dispersion changes across the entire human lifespan. Third, we used the Yeo-7 functional atlas to validate our results, and found a similar tendency across the human lifespan. However, future studies should identify brain regions or networks that have been highly implicated in specific cognitive processes, such as the theory of mind and empathy [60], sustained attention [61], and encoding of emotion concepts [62]. In addition, the human brain has less functional connectivities within networks and greater connectivities between networks during aging, which helps to sustain cognitive function and is a typical response to aging [63]. Thus, the capacity to maintain connectivities between networks might protect brain health [64]. Cognitive reserve is another similar possibility. Cognitive training and engagement in social activities may enhance brain network connectivity to increase brain resilience [65]. The current age-related changes in the MSN manifold showed a standardized assessment of multiple morphometric features in atypical brains, providing new insights into the influence of such changes on cognitive performance. Finally, we investigated the relationships between age-related changes in dispersion and molecular factors. The AHBA gene data and neurotransmitter receptor data came from separate cohorts, limiting the examination of molecular-neuroimaging associations across the entire human lifespan, and possibly omitting individual effects.

In summary, we identified age-related changes in global, within-, and between-network dispersions in a multidimensional gradient framework using morphometric similarity organization. Our findings revealed that associations and secondary sensory cortices showed an expansion and contraction of their corresponding connectome manifold signatures, respectively. The cytoarchitecture communities themselves were increasingly dispersed with increasing age and could mediate the effect on age-related cognitive flexibility of EFs decline. Our multidimensional framework described within-network disassortativity and between-network assortativities in the brain, enriching our understanding of developmental characterization.

## Methods

### Study participants

We included normal healthy participants from childhood to advanced ages. We used 3 neuroimaging datasets (total *n* = 1,790) publicly available from Lifespan Human Connectome Project (HCP) in Development (hereafter, HCP-D, age range: 8 to 21 years), in Young Adult (HCP-YA, age range: 22 to 35 years), and in Aging (HCP-A, age range: 36 to 100 years) [66]. In accordance with the Declaration of Helsinki, all participants gave written informed consent. Participants under 18 years were accompanied by a parent or legal guardian who gave informed, written permission for their child’s participation. For the HCP dataset, ethical approval was given by the Washington University Institutional Review Board (IRB #201204036).

#### HCP-D

The samples of normally developing children and adolescents were scanned in Boston, Los Angeles, Minneapolis, and St. Louis [67]. To ensure the validity of samples, individuals were excluded if they were born prematurely, required special educational services, had MRI contraindications, or had a history of serious medical problems, head injury, endocrine disorders, psychiatric disorders, or neurodevelopmental disorders. Among the initially considered participants, 19 participants were excluded because they were younger than 8 years. As a result, the present analyses included 633 participants (339 females) with 3T scanning neuroimaging data from the HCP-D 2.0 Release. The included participants had ages between 8.08 and 21.92 years, with a mean age of 14.65 ± 3.90 years.

#### HCP-YA

The sample of healthy young adults was obtained through the WU-Minn Human Connectome Project at Washington University in St. Louis [68]. Participants in HCP-D were screened by following the exclusionary criteria of HCP-YA, and additionally, individuals with mini-mental state examination scores below 25 were excluded. At the time of the present study, neuroimaging data were available for 1,113 participants from the HCP S1,200 Release. Because the HCP-YA included twins and non-twin siblings, only 1 participant from each family was randomly selected, resulting in 445 participants (239 females). The included participants had ages between 22 and 36 years, with a mean age of 28.64 ± 3.73 years.

#### HCP-A

The sample of cognitively normal aging adults, older than 36, was recruited and scanned at Washington University St Louis, University of Minnesota, Massachusetts General Hospital, and the University of California Los Angeles [69]. Like the exclusionary criteria used in HCP-D and HCP-YA, individuals with impaired cognitive abilities were excluded from HCP-A based on tiered age-appropriate cut-off scores. At the time of analysis, there were 725 participants with available neuroimaging data from the HCP-A 2.0 Release. Individuals were excluded if they were over 89 years old, resulting in 712 participants (406 females). The included participants had ages between 36.00 and 89.86 years, with a mean age of 60.36 ± 14.88 years.

### MRI acquisition

The participants’ MRI data were obtained using different 3-T scanners and acquisition parameters based on the cohort they belonged to. All MRI data are available at HCP’s Connectome Database (https://www.humanconnectome.org/software/connectomedb).

For the initial HCP-YA dataset, participants were scanned on a Customized Siemens 3T Connectome Skyra (Siemens Healthineers) with a 100 mT gradient coil and a 32-channel head coil. The T1w images were acquired using a 3D single-echo magnetization prepared rapid gradient echo (MP-RAGE) sequence with the following parameters: repetition time (TR) = 2,400 ms, echo time (TE) = 2.14 ms, inversion time (TI) = 1,000 ms, flip angle (FA) = 8°, field of view (FOV) = 224 × 224 mm^2^, matrix = 320 × 320, voxel size = 0.7 mm isotropic, with 256 sagittal slices.

For HCP-D and HCP-A datasets, participants were scanned on Siemens PRISMA (Siemens Healthineers) with an 80 mT/m gradient coil and a 32-channel head coil. Slight variations were made in the acquisition parameters to account for the unique challenges of working with younger and older populations [70]. Scans for the HCP-D and HCP-A participants were performed on a variant of the HCP-YA Connectome scanner, the Siemens 3T Prisma, equipped with the T1w multi-echo MP-RAGE scans that were acquired with the following parameters: 4 echoes per line of k-space, TR = 2,500 ms, TI = 1,000 ms, TE = 1.8/3.6/5.4/7.2 ms, FA = 8°, FOV = 256 × 240 × 166 mm^3^, matrix = 320 × 300 × 208 slices, and voxel size = 0.8 mm isotropic. These acquisition parameters slightly differed from those in the HCP-YA, which accounted for the unique challenges of working with younger and older populations [70].

### MRI data processing

All 3D T1w data were preprocessed using the HCP minimal processing pipeline [71]. Brain extraction and readout distortion correction using a field map were then conducted. Anatomical surfaces were generated from the individual T1w image in native space, then reconstruction of the gray/white interface and the pial surface was conducted. Five morphometric features were obtained from the individual surface, including gray matter volume (GMV), surface area (SA), cortical thickness (CT), Gaussian curvature (GC), and mean curvature (MC) [27,37–39].

### MSN construction

The cortical D-K atlas was divided into 1,533 spatially contiguous regions of approximately 1 cm^2^ size [27,72]. For each participant, 5 morphometric feature vectors were z-normalized across regions to account for variations in value distributions between features. Pearson’s correlation coefficients were calculated based on the morphometric features between each paired cortical region, forming a 1,533 × 1,533 morphometric similarity matrix [27,38]. In addition, we used 2 more features (i.e., fractional anisotropy and mean diffusivity) derived from diffusion-weighted images to measure the robustness of the MSN matrix.

### Manifold construction of the MSN

To identify spatial axes (connectivity gradients) of inter-regional morphometric variations, we performed morphometric similarity manifolds analyses using the BrainSpace toolbox [73]. First, we utilized row-wise thresholding to maintain the top 10% connections per row, and then computed the cosine distance between each row to produce an affinity matrix that effectively captured similarities in the profiles of the MSN [27,74–76]. Second, we used diffusion map embedding [23] and a nonlinear dimensionality reduction technique, to identify principal gradient components, explaining connectivity variations in descending order. Following recommended guidelines [77], we set the manifold parameters to a = 0.5 and t = 0, ensuring the preservation of global relations between data points within the embedded space. To provide the basis for comparing changes in the MSN across the human lifespan, we constructed a group manifold from the averaged MSN matrix across all participants (after harmonizing MSN to remove site effects). This group manifold underwent the same aforementioned manifold construction procedures, and as such, we aligned all individuals’ MSN manifolds to the group manifold via Procrustes rotations [78]. All follow-up analyses on the aligned manifolds were performed using the top 3 gradients.

### The 3D gradient dispersions

We computed the Euclidean distance between a single region and the manifold centroid as global dispersion [14,28,78,79] at an individual level. It relates to eccentricity and provided a scalar index of whole-brain integration and segregation. The distal regions with greater global dispersion were more segregated than proximal regions that were more broadly integrated. Within-network dispersion was quantified as the sum squared Euclidean distance of each region’s gradient score from a specific network centroid [28,79,80]. It thus captured the similarity of MSN profiles within a given network. Lower values suggested more uniform MSN patterns within the network. Between-network dispersion was calculated as the Euclidean distance between network centroids, which provided a measure of the differentiation of 2 given networks from one another. Lower values suggested less differentiated MSN patterns across different networks. Lower within-network/between-network dispersion values were described as segregation of within or between networks. This analysis used the Von Economo atlas-defined 7 cytoarchitectural classes.

### Age was associated with 3D gradient dispersions

We constructed an association model with lifespan age and gradient dispersion. Before fitting the association, we first removed multi-site (scanner) effects on dispersions [81] using the ComBat technique [31,82]. Based on our previous study [28], GAMLSS calculated in statistical software R [83] was used to estimate the association between ages and brain dispersions, including global, within-, and between-network dispersions. GAMLSS used a penalized maximum likelihood approach to estimate smoothness parameters (effective degrees of freedom), which were subsequently used to estimate the μ and σ parameters. The adequacy of fit for these parameters in the GAMLSS algorithm was determined by minimizing the generalized Akaike information criterion (GAIC) index. Each fitting curve was modeled using 1 distribution according to the GAIC to estimate nonlinear normative growth curves. Age was included as a continuous variable in the models, with sex and eTIV as covariates. To identify developmental windows of brain significant dispersion, we measured the first derivative of the smooth age term, which indicates the change of spline fit at every age. We operationalized the window of significant age-related change as the period at which the 95% point-wise CI of the spline’s estimated slope did not include 0 [32,33]. According to a previous study [84], we evaluated the age effects (R^2^) in each GAMLSS model. The effect size of age spline is calculated as the change in generalized (Pseudo) R-square, which is the proportion of variance explained by a full model that is not explained by a reduced model, using the Rsq function in the GAMLSS model. Because R^2^ describes effect size but not direction (i.e., increasing or decreasing brain dispersion with age), we extracted and applied the sign of the age coefficient from nonlinear model [32,33].

### Structural equation modeling

A cognitive variable representing an index of EF (both fluid intelligence and multi-tasking) can be used to predict brain imaging [34,85]. These conjectures of brain and behavior relationships were then related to age-related declines in EFs [86–88]. Thus, modeling of more specific neural properties may be necessary to inform neurocognitive theories of aging [89,90]. We used structural equation modeling (SEM), a powerful multivariate technique that fits observed covariances between variables, to identify age-related changes in multi-networks dispersions. The SEM was fit using maximum likelihood estimation and passed the bootstrap test via the *Lavaan* package in R. The mediating influence values of multiple mediations were calculated as follows:

$$\frac{\left(a1\times b1\right)+\left(a2\times b2\right)+\cdots +\left(a\text{n}\times b\text{n}\right)}{\left(a1\times b1\right)+\left(a2\times b2\right)+\cdots +\left(a\text{n}\times b\text{n}\right)+c\prime }.$$

The simple middle of a mediating variable is depicted in the following equation:

$$\frac{\left(a\times b\right)}{\left(a\times b\right)+c\prime }.$$

#### Molecular fingerprints underpinning age-dispersion association

*Brain-wide gene expression profiles*. We identified the potential biological explanations of associations with age-related changes in global dispersions at different biological scales. We first analyzed its consistency with spatial variations of the whole brain gene expression system [91]. The microarray expression data were obtained from 6 postmortem brains provided by the AHBA (http://human.brain-map.org/) [92]. Genetic data were preprocessed with the *abagen* toolbox (https://github.com/netneurolab/abagen) using the D-K1533 atlas. Briefly, probes were reannotated using data provided by Arnatkeviciute and colleagues [93] and filtered based on an expression intensity-based threshold of 50%. Samples were assigned to brain regions within 2 mm of a given parcel. If a brain region was not assigned a sample from any donor based on the above procedure, the tissue sample closest to the centroid of that parcel was independently identified for each donor. The average of these samples was taken across all donors, and weighted by the distance between the parcel centroid and the sample, to estimate the parcellated expression values for the missing regions. Gene expression values were then normalized across tissue samples using an identical procedure. Considering that the AHBA dataset included only 2 right hemisphere data, only the left hemisphere was considered in our analysis. Samples assigned to the same brain region were averaged, yielding a regional expression with 767 regions and 15,632 genes. A threshold of 0.1 was used to further filter the stable genes on the differential stability of each gene, resulting in 7,645 retained genes.

The PLS regression analysis [94] was used to investigate the relationships between developmental changes in global dispersions (R^2^-values obtained from 767 cortical regions in the left hemisphere) and transcriptional activity for all 7,645 genes. Gene expressions data were served as predictor variables for developmental changes in global dispersions within the PLS regression framework. The first PLS (PLS1) component represented a linear combination of gene expression values that exhibited the strongest correlation with developmental changes in global dispersions. Age was permutated and the relationships between global dispersions and PLS regression analysis were recalculated 1,000 times to test whether the actual correlation was greater than the permutated associations. Bootstrap resampling was used to estimate the variability of each gene’s PLS1. The ratio of the weight of each gene to its bootstrap standard error was calculated to determine the z-scores and rank the genes according to their contributions to PLS1 [40]. The top 1,000 genes constituted the developmental changes in the global dispersion gene list.

The top 1,000 significant gene list was subjected to enrichment analysis using the CSEA developmental express tool (http://genetics.wustl.edu/jdlab/csea-tool-2) [95]. The CSEA aims to investigate the neurodevelopmental impact of genes within a given set by analyzing their involvements in the formation and differentiation of specific brain structures, such as the amygdala, cerebellum, cortex, hippocampus, striatum, and thalamus, across various developmental stages, ranging from early fetal life to young adulthood. Significance was calculated based on Fisher’s exact test with FDR correction.

#### Neurotransmitter receptors profiles

The brain-wide neurotransmitter receptor densities were estimated using 18 PET-derived tracer images from Hansen and colleagues [36] (https://github.com/netneurolab/hansen_receptors) and Markello and colleagues [96] (neuromaps, v0.0.1, https://github.com/netneurolab/neuromaps). These receptors/transporters combined into 8 neurotransmitter systems as follows: dopamine (D1, D2, DAT), norepinephrine (NET), serotonin (5-HT1A, 5-HT1B, 5-HT2, 5-HT4, 5-HT6, 5-HTT), acetylcholine (α4β2, M1, VAChT), glutamate (mGluR5), GABA (GABAa), histamine (H3), and cannabinoid (CB1) [97]. We re-warped the cortical DK1533 parcellation onto volumetric PET image space. Next, neurotransmitter receptor profiles were parcellated to 1,533 brain regions and z-scored, resulting in a 1,533 regions × 8 receptors matrix of relative densities.

A multivariate linear regression model combining 8 neurotransmitter receptors was used to identify the molecular contributions to age-related changes in global dispersions [98,99]. The age-related changes in global dispersion (R^2^-values) as responder and receptor matrix as predictors were included into the *relaimpo* package (relative importance of regressors in linear models, version 2.2–5) in R. Relative importance metrics can be used to address linear regression with multiple collinear regressors.

## Supporting information

S1 Fig Flowchart of the study participants. (PDF)

S2 Fig Sample age distributions. (PDF)

S3 Fig Spatial correspondence between the mean MSN matrices across subjects derived from 5 and 7 features (*r* = 0.74, *P* < 0.001). (PDF)

S4 Fig Global dispersion with age based on the MSN matrix derived from 7 features. (PDF)

S5 Fig Global dispersion with age for 4 age windows. (PDF)

S6 Fig Within-network dispersion with age for 4 age windows. (PDF)

S7 Fig Within-network dispersion with age based on Yeo-7 functional atlas. (PDF)

S8 Fig Between-network dispersion with age for 4 age windows. (PDF)

S9 Fig Between-network dispersion with age based on Yeo-7 functional atlas. (PDF)

S1 Table The type of modality and morphometric features in constructing MSN. (PDF)

S2 Table Results of multiple linear regression model relating neurotransmitter receptors and age-related changes in regional manifold eccentricity. (PDF)

S3 Table Age-related differences in within-network dispersion, controlling for sex and eTIV. (PDF)

S4 Table Age-related differences in within-network dispersion for 4 age windows, controlling for sex and eTIV. All *p* values were corrected by FDR.
(PDF)

S5 Table Age-related changes in within-network dispersion medicated the influence of age on cognitive flexibility of executive function. (PDF)

S6 Table Age-related differences in between-network dispersion, controlling for sex and eTIV. All *p* values were corrected by FDR.
(PDF)

S7 Table Age-related differences in between-network dispersion for 4 age windows, controlling for sex and eTIV. All *p* values were corrected by FDR.
(PDF)

S1 Data Data underlying figures except for S3 Fig. (XLSX)

S2 Data Data underlying x-axis of S3 Fig. (XLSX)

S3 Data Data underlying y-axis of S3 Fig. (XLSX)

## Abbreviations

CI: confidence interval
CSEA: cell type-specific expression analysis
CT: cortical thickness
EFs: executive functions
eTIV: estimated total intracranial volume
FA: flip angle
FDR: false discovery rate
FOV: field of view
GAIC: generalized Akaike information criterion
GALSS: generalized additive model for location, scale, and shape
GC: Gaussian curvature
GMV: gray matter volume
HCP-A: Human Connectome Project in Aging
HCP-D: Human Connectome Project in Development
HCP-YA: Human Connectome Project in Young Adult
MC: mean curvature
MP-RAGE: magnetization prepared rapid gradient echo
MRI: magnetic resonance imaging
MSN: morphometric similarity network
PET: positron emission tomography
PLS: partial least squares
SA: surface area
SCN: structural covariance network
SEM: structural equation modeling
TE: echo time
TI: inversion time
TR: repetition time
