# Supplementary figures and images for: Morphometric brain organization across the human lifespan reveals increased dispersion linked to cognitive performance
Source: PLoS Biol. 2024 Jun 20;22(6):e3002647. doi: 10.1371/journal.pbio.3002647 (PMC11189252; doi:10.1371/journal.pbio.3002647)

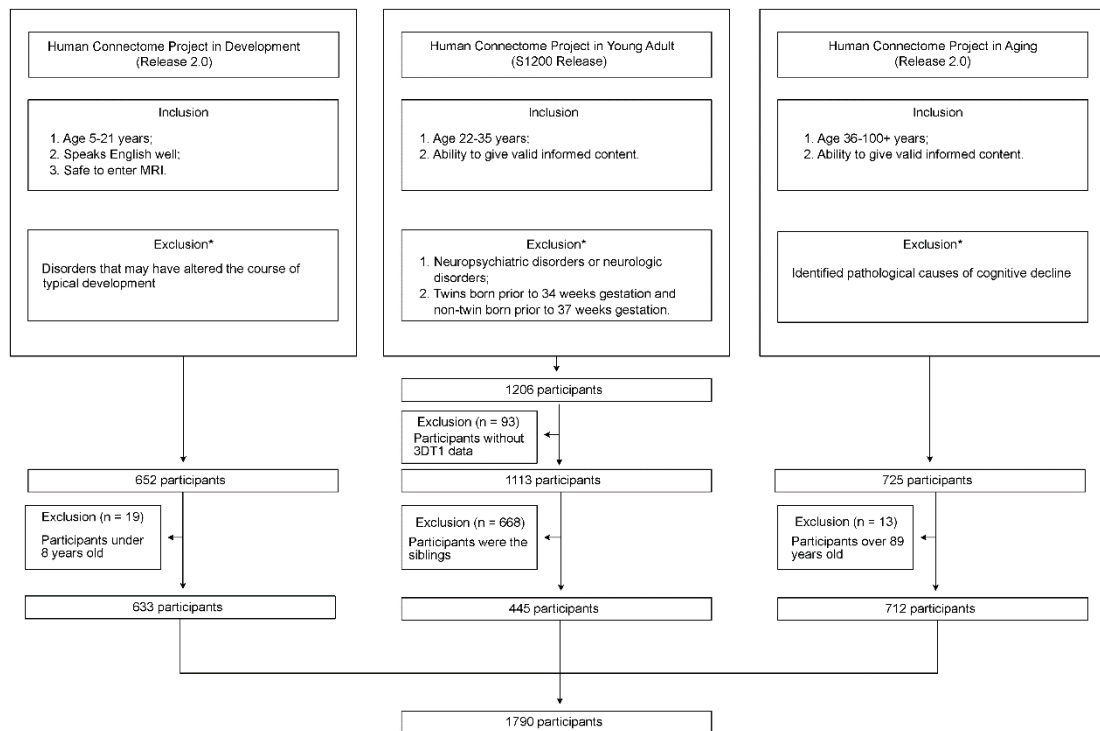

**Figure S1. Flowchart of the study participants.**

Supplement: S1 Fig — (PDF) [file pbio.3002647.s001.pdf]
