# Supplementary material for: Morphometric brain organization across the human lifespan reveals increased dispersion linked to cognitive performance
Source: PLoS Biol. 2024 Jun 20;22(6):e3002647. doi: 10.1371/journal.pbio.3002647 (PMC11189252; doi:10.1371/journal.pbio.3002647)
Supplement: S2 Fig — (PDF) [file pbio.3002647.s002.pdf]

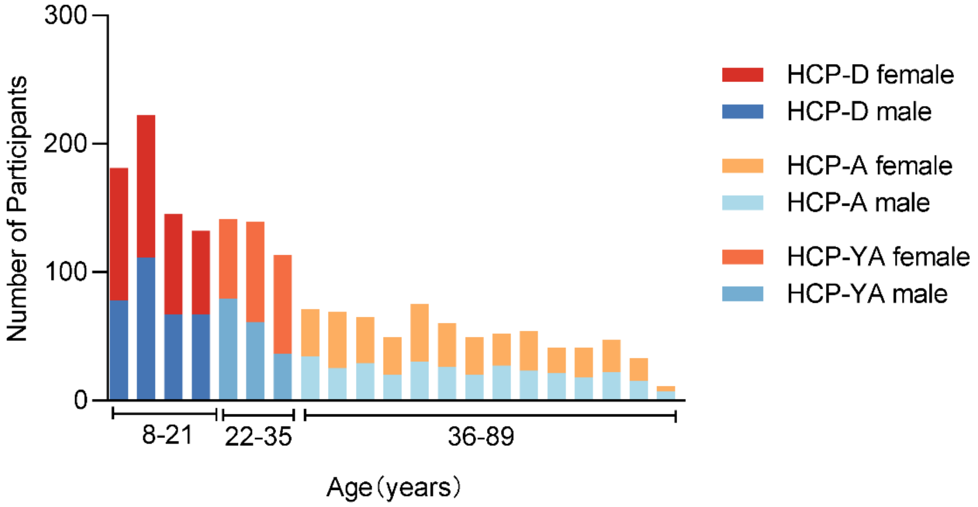

Figure S2. Sample age distributions. Each bin includes four age intervals. The data underlying this figure can be found in S1 data.
