# Supplementary material for: Morphometric brain organization across the human lifespan reveals increased dispersion linked to cognitive performance
Source: PLoS Biol. 2024 Jun 20;22(6):e3002647. doi: 10.1371/journal.pbio.3002647 (PMC11189252; doi:10.1371/journal.pbio.3002647)
Supplement: S3 Fig — (PDF) [file pbio.3002647.s003.pdf]

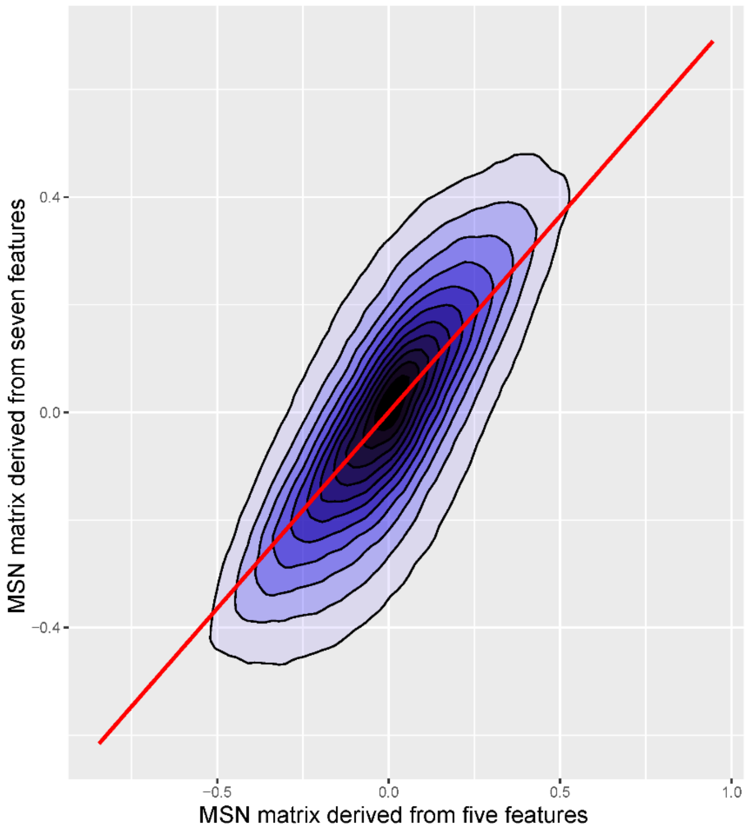

Figure S3. Spatial correspondence between the mean MSN matrices across subjects derived from five and seven features (*r* = 0.74, *P* < 0.001). The data underlying this figure can be found in S2 (x-axis) and S3 (y-axis) data.
