# Supplementary material for: Morphometric brain organization across the human lifespan reveals increased dispersion linked to cognitive performance
Source: PLoS Biol. 2024 Jun 20;22(6):e3002647. doi: 10.1371/journal.pbio.3002647 (PMC11189252; doi:10.1371/journal.pbio.3002647)
Supplement: S5 Fig — (PDF) [file pbio.3002647.s005.pdf]

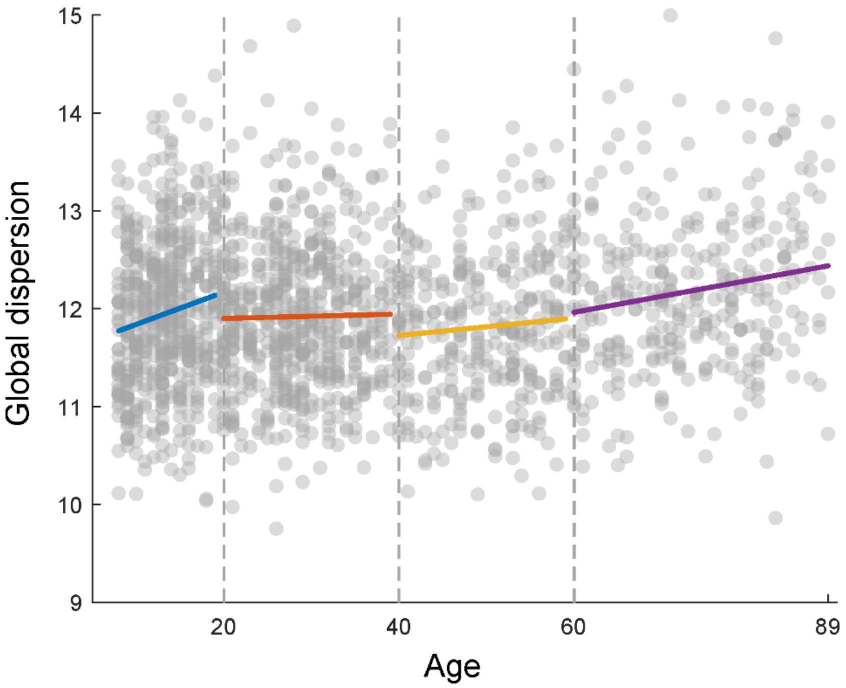

Figure S5. Global dispersion with age for four age windows. Participants were segmented into four age windows (late childhood to adolescence: 8−19 years; young adulthood: 20−39 years; middle adulthood: 40−59 years; and late adulthood: 60−89 years). Significant increases were observed in global dispersions during adolescence (t = 2.97, P_{FDR} = 0.01) and late adulthood (t = 2.81, P_{FDR} = 0.01). The data underlying this figure can be found in S1 data.
