# Supplementary material for: Morphometric brain organization across the human lifespan reveals increased dispersion linked to cognitive performance
Source: PLoS Biol. 2024 Jun 20;22(6):e3002647. doi: 10.1371/journal.pbio.3002647 (PMC11189252; doi:10.1371/journal.pbio.3002647)
Supplement: S7 Fig — (PDF) [file pbio.3002647.s007.pdf]

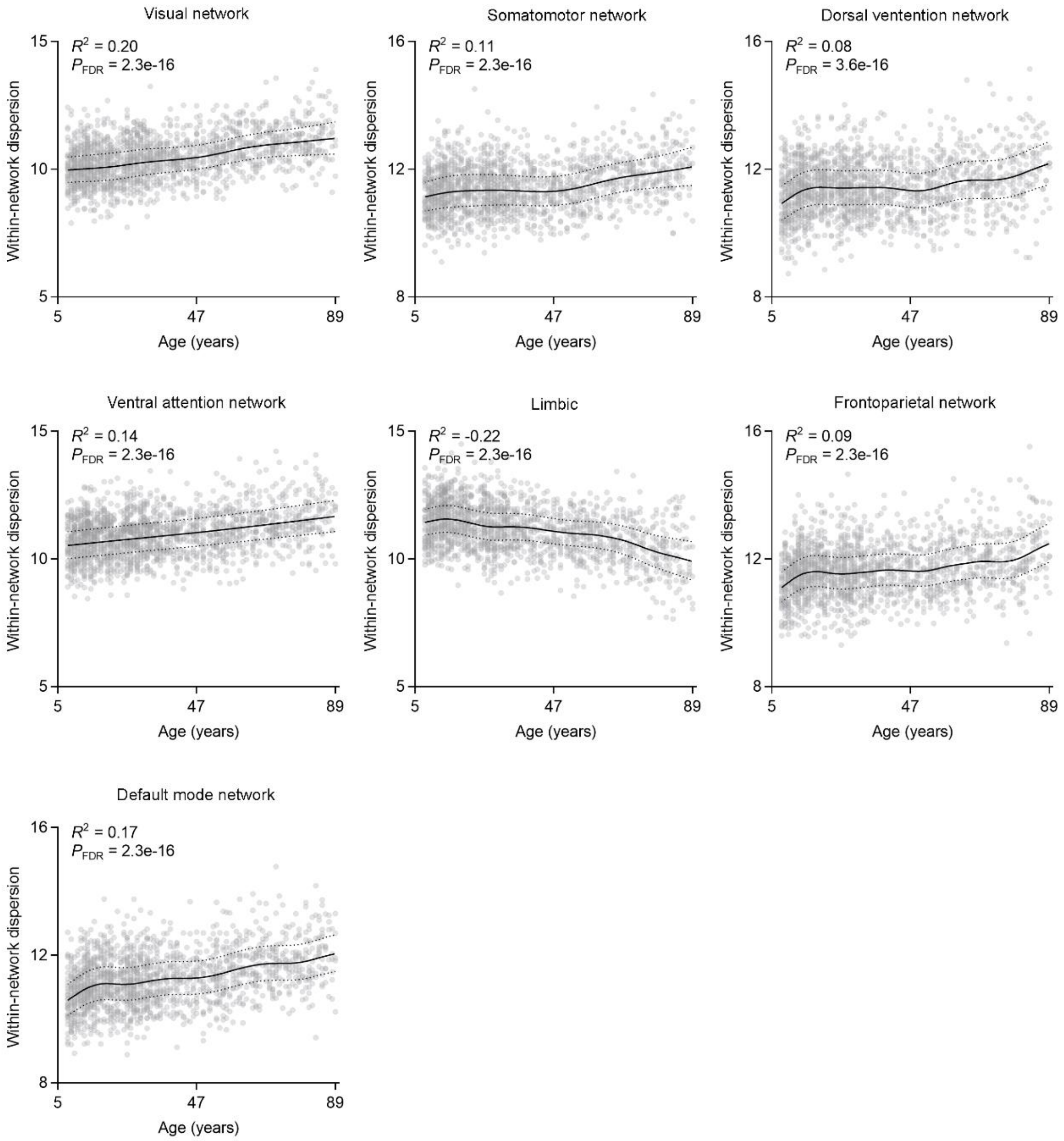

Figure S7. Within-network dispersion with age based on Yeo-7 functional atlas. *P* values were corrected by the false-discovery rate. The data underlying this figure can be found in S1 data.
