# Supplementary material for: Morphometric brain organization across the human lifespan reveals increased dispersion linked to cognitive performance
Source: PLoS Biol. 2024 Jun 20;22(6):e3002647. doi: 10.1371/journal.pbio.3002647 (PMC11189252; doi:10.1371/journal.pbio.3002647)
Supplement: S1 Table — (PDF) [file pbio.3002647.s010.pdf]

Table S1. The type of modality and morphometric features in constructing MSN.

No. of features	Modality	Features	Study
12	*T1w, T2w, DWI*	*GMV, T1w/T2w, FA, MD, RD, AD, kurtosis, IVF, isVF, OD, OOD*	*Galdi et al, 2020* [1]
10	*T1w, DWI*	*GMV, SA, CT, FA, MD, MT, IC, MC, CI, FI*	*Seidlitz et al., 2018* [2]
(1) 7<br>*(2) 8*<br>*(3) 10*	*(1) T1w*<br>*(2) T1w, T2w*<br>*(3) T1w, T2w, DWI*	*(1) GMV, SA, CT, IC, MC, CI, FI*<br>*(2) GMV, SA, CT, IC, MC, CI, FI, T1w/T2w*<br>*(3) GMV, SA, CT, FA, MD, IC, MC, CI, FI, T1w/T2w*	*King et al., 2020* [3]
9	*T1w*	*number of vertices, GMV, SA, CT, stdCT, MC, IC, CI, FI*	*Li et al., 2017* [4]
7	*T1w*	*CT, SA, GMC, LGI, SD, GH, CI*	*Zheng et al, 2019* [5]
7	*T1w*	*SA, GMV, CT, CI, stdCT, CI, FI*	*RaviPrakash et al., 2021* [6]
7	*T1w*	*GMV, SA, CT, IC, MC, CI, FI*	*Vasa et al., 2022* [7]
7	*T1w, DWI*	*GMV, SA, CT, IC, MC, FA, MD*	*Morgan et al., 2019* [8]
7	*T1w*	*GMV, SA, CT, IC, MC, FI, CI*	*Zhukovsky et al., 2022* [9]
7	*T1w*	*GMV, SA, CT, IC, MC, FI, CI*	*Li et al., 2023* [10]
6	*T1w*	*CT, SA, GMV, LGI, SD, GH*	*Zheng et al, 2018* [11]
5	*T1w*	*CT, SA, GMV, MC, IC*	*Seidlitz et al., 2020* [12]
5	*T1w*	*CT, SA, GMV, MC, IC*	*Yang et al, 2021* [13]
5	*T1w, DWI, fMRI*	*CT, MD, FA, FC, SC*	*Morgan et al., 2021* [14]
5	*T1w*	*CT, SA, GMV, MC, IC*	*Long et al., 2023* [15]
5	*T1w*	*GMV, SA, CT, IC, MC*	*The present work*

Abbreviations: CI, curvature index; CT, cortical thickness; DWI, diffusion weighted imaging; FA, fractional anisotropy; FI, folding index; fMRI, Functional magnetic resonance imaging; GH, gyrus height; GMV, gray-matter volume; IC, intrinsic (Gaussian) curvature; isVF, isotropic volume fraction; IVF, intracellular volume fraction; LGI, local gyrification index; MC, mean curvature; MD, mean diffusivity; MT, magnetization transfer; OD, orientation dispersion index along the primary and secondary directions; OOD, overall orientation dispersion index; RD, radial diffusivity; SA, surface area; SD, sulcal depth; stdCT, standard error of cortical thickness; T1w, T1 weighted image; T2w, T2 weighted image.

Supplementary References

1. Galdi, P, Blesa, M, Stoye, DQ, Sullivan, G, Lamb, GJ, Quigley, AJ et al. Neonatal morphometric similarity mapping for predicting brain age and characterizing neuroanatomic variation associated with preterm birth. Neuroimage Clin. 2020; 25: 102195.
2. Seidlitz, J, Vasa, F, Shinn, M, Romero-Garcia, R, Whitaker, KJ, Vertes, PE et al. Morphometric similarity networks detect microscale cortical organization and predict inter-individual cognitive variation. Neuron. 2018; 97: 231-247 e237.
3. King, DJ, Wood, AG Clinically feasible brain morphometric similarity network construction approaches with restricted magnetic resonance imaging acquisitions. Netw Neurosci. 2020; 4: 274-291.
4. Li, W, Yang, C, Shi, F, Wu, S, Wang, Q, Nie, Y et al. Construction of individual morphological brain networks with multiple morphometric features. Front Neuroanat. 2017; 11: 34.
5. Zheng, W, Eilam-Stock, T, Wu, T, Spagna, A, Chen, C, Hu, B et al. Multi-feature based network revealing the structural abnormalities in autism spectrum disorder. IEEE Trans Affect Comput. 2019; 12: 732-742.
6. RaviPrakash, H, Anwar, SM, Biassou, NM, Bagci, U Morphometric and functional brain connectivity differentiates chess masters from amateur players. Front Neurosci. 2021; 15: 629478.
7. Vasa, F, Hobday, H, Stanyard, RA, Daws, RE, Giampietro, V, O'Daly, O et al. Rapid processing and quantitative evaluation of structural brain scans for adaptive multimodal imaging. Hum Brain Mapp. 2022; 43: 1749-1765.
8. Morgan, SE, Seidlitz, J, Whitaker, KJ, Romero-Garcia, R, Clifton, NE, Scarpazza, C et al. Cortical patterning of abnormal morphometric similarity in psychosis is associated with brain expression of schizophrenia-related genes. Proc Natl Acad Sci U S A. 2019; 116: 9604-9609.
9. Zhukovsky, P, Savulich, G, Morgan, S, Dalley, JW, Williams, GB, Ersche, KD Morphometric similarity deviations in stimulant use disorder point towards abnormal brain ageing. Brain Commun. 2022; 4: fcac079.
10. Li, J, Keller, SS, Seidlitz, J, Chen, H, Li, B, Weng, Y et al. Cortical morphometric vulnerability to generalised epilepsy reflects chromosome- and cell type-specific transcriptomic signatures. Neuropathol Appl Neurobiol. 2023; 49: e12857.
11. Zheng, W, Yao, Z, Xie, Y, Fan, J, Hu, B Identification of Alzheimer's disease and mild cognitive impairment using networks constructed based on multiple morphological brain features. Biol Psychiatry Cogn Neurosci Neuroimaging. 2018; 3: 887-897.
12. Seidlitz, J, Nadig, A, Liu, S, Bethlehem, RAI, Vertes, PE, Morgan, SE et al. Transcriptomic and cellular decoding of regional brain vulnerability to neurogenetic disorders. Nat Commun. 2020; 11: 3358.
13. Yang, S, Wagstyl, K, Meng, Y, Zhao, X, Li, J, Zhong, P et al. Cortical patterning of morphometric similarity gradient reveals diverged hierarchical organization in

sensory-motor cortices. Cell Rep. 2021; 36: 109582.
14. Morgan, SE, Young, J, Patel, AX, Whitaker, KJ, Scarpazza, C, van Amelsvoort, T et al. Functional magnetic resonance imaging connectivity accurately distinguishes cases with psychotic disorders from healthy controls, based on cortical features associated with brain network development. Biol Psychiatry Cogn Neurosci Neuroimaging. 2021, 6: 1125-1134.
15. Long, J, Li, J, Xie, B, Jiao, Z, Shen, G, Liao, W et al. Morphometric similarity network alterations in COVID-19 survivors correlate with behavioral features and transcriptional signatures. Neuroimage Clin. 2023; 39: 103498.
