# Supplementary material for: Morphometric brain organization across the human lifespan reveals increased dispersion linked to cognitive performance
Source: PLoS Biol. 2024 Jun 20;22(6):e3002647. doi: 10.1371/journal.pbio.3002647 (PMC11189252; doi:10.1371/journal.pbio.3002647)
Supplement: S2 Table — (PDF) [file pbio.3002647.s011.pdf]

Table S2. Results of multiple linear regression model relating neurotransmitter receptors and age-related changes in regional manifold eccentricity.

Metrics	β-value	Standard error	T-value	Relative importance (%)	P_{spin}-value FDR-corrected
Intercept	0.015	0.002	7.5	N.A.	N.A.
Serotonin	-0.007	0.003	-2.30	2.4	0.38
Dopamine	-0.031	0.004	-8.40	9.7	0.07
Histamine	0.017	0.003	5.40	5.2	0.21
Acetylcholine	-0.019	0.002	-8.76	31.7	0.003
Cannabinoid	-0.008	0.003	-2.92	1.7	0.35
Glutamate	0.045	0.003	15.88	45.5	0.07
GABA	0.01	0.003	3.58	2.9	0.38
Noradrenaline	0.006	0.003	2.48	0.9	0.33
