# Supplementary material for: Morphometric brain organization across the human lifespan reveals increased dispersion linked to cognitive performance
Source: PLoS Biol. 2024 Jun 20;22(6):e3002647. doi: 10.1371/journal.pbio.3002647 (PMC11189252; doi:10.1371/journal.pbio.3002647)
Supplement: S3 Table — (PDF) [file pbio.3002647.s012.pdf]

Table S3. Age-related differences in within-network dispersion, controlling for sex and eTIV.

von Economo class	R^2 values	β	se	P_{FDR} value
Primary motor	0.22	1.92e-02	9.79e-04	<2.8e-16
Association 1	0.10	8.29e-04	6.88e-05	<2.8e-16
Association 2	0.15	1.17e-03	7.15e-05	<2.8e-16
Secondary sensory	0.15	1.24e-03	7.86e-05	<2.8e-16
Primary sensory	0.06	2.94e-03	1.03e-03	0.00413
Limbic	0.04	5.35e-03	1.06e-03	6.29e-07
Insular	0.21	2.46e-02	1.43e-03	<2.8e-16
