# Supplementary material for: Morphometric brain organization across the human lifespan reveals increased dispersion linked to cognitive performance
Source: PLoS Biol. 2024 Jun 20;22(6):e3002647. doi: 10.1371/journal.pbio.3002647 (PMC11189252; doi:10.1371/journal.pbio.3002647)
Supplement: S4 Table — All p values were corrected by FDR. (PDF) [file pbio.3002647.s013.pdf]

Table S4. Age-related differences in within-network dispersion for four age windows, controlling for sex and eTIV. All *p* values were corrected by FDR.

	Adolescence	Young Adulthood	Middle Adulthood	Late Adulthood
Primary motor	t = -2.75*, p = 0.01	t = 2.70*, p = 0.01	t = 3.67*, p = 0.0009	t = 5.16*, p = 4e-6
Association1	t = 5.01*, p = 5e-6	t = 1.61, p = 0.14	t = 1.45, p = 0.17	t = 3.34*, p = 0.003
Association2	t = 7.22*, p = 5e-11	t = 2.05, p = 0.06	t = 2.19*, p = 0.05	t = 2.89*, p = 0.009
Secondary sensory	t = -1.14, p = 0.28	t = 3.68*, p = 0.0009	t = 2.71*, p = 0.01	t = 2.92*, p = 0.0087
Limbic	t = 5.67*, p = 3e-7	t = 1.52, p = 0.16	t = 0.34, p = 0.73	t = -1.52, p = 0.16
Primary sensory	t = 1.72, p = 0.12	t = 3.8*, p = 6e-4	t = 1.43, p = 0.17	t = 0.58, p = 0.58
Insular	t = 2.33*, p = 0.03	t = 4.35*, p = 9e-5	t = 2.96*, p = 0.008	t = 3.99*, p = 0.0003
