# Supplementary material for: Morphometric brain organization across the human lifespan reveals increased dispersion linked to cognitive performance
Source: PLoS Biol. 2024 Jun 20;22(6):e3002647. doi: 10.1371/journal.pbio.3002647 (PMC11189252; doi:10.1371/journal.pbio.3002647)
Supplement: S5 Table — (PDF) [file pbio.3002647.s014.pdf]

Table S5. Age-related changes in within-network dispersion medicated the influence of age on cognitive flexibility of executive function.

**Path a**	z	β	se	P_{FDR} value
Primary motor	16.73	0.019	0.001	0
Association1	9.65	0.009	0.001	0
Association2	13.51	0.013	0.001	0
Secondary sensory	13.84	0.013	0.001	0
Primary sensory	2.02	0.002	0.001	0.04
Limbic	3.23	0.004	0.001	0.001
Insular	14.47	0.03	0.002	0
**Path b**				
Primary motor	-3.71	-1.40	0.38	0
Association1	-0.40	-0.41	1.02	0.69
Association2	3.08	2.67	0.87	0.007
Secondary sensory	-1.74	-0.81	0.47	0.15
Primary sensory	-0.53	-0.24	0.45	0.69
Limbic	-0.45	-0.15	0.34	0.69
Insular	-1.88	-0.41	0.22	0.14
**Indirect effect (a × b)**				
Primary motor	-3.64	-0.03	0.007	0
Association1	-0.40	-0.004	0.009	0.69
Association2	3.01	0.03	0.011	0.008
Secondary sensory	-1.71	-0.01	0.006	0.12
Primary sensory	-0.46	-0.001	0.001	0.69
Limbic	-0.42	-0.001	0.001	0.69
Insular	-1.86	-0.01	0.005	0.11
Indirect effect	-1.99	-0.02	0.009	0.04
Direct effect	-4.81	-0.08	0.016	0
Total effect	-7.20	-0.09	0.013	0
