# Supplementary material for: Morphometric brain organization across the human lifespan reveals increased dispersion linked to cognitive performance
Source: PLoS Biol. 2024 Jun 20;22(6):e3002647. doi: 10.1371/journal.pbio.3002647 (PMC11189252; doi:10.1371/journal.pbio.3002647)
Supplement: S6 Table — All p values were corrected by FDR. (PDF) [file pbio.3002647.s015.pdf]

Table S6. Age-related differences in between-network dispersion, controlling for sex and eTIV. All *p* values were corrected by FDR.

	Primary motor	Association1	Association2	Secondary sensory	Primary sensory	Limbic
Association1	R^2 = 0.08*; *p* = 6.05e-15					
Association2	R^2 =0.06; *p* = 0.26	R^2 = 0.02*; *p* = 0.004				
Secondary sensory	R^2 = -0.25*; *p* = 3.82e-16	R^2 =-0.38*; *p* <3.82e-16	R^2 = -0.29*; *p* < 3.82e-16			
Primary sensory	R^2 = -0.14*; *p* < 3.8e-16	R^2 = -0.07*; *p* = 0.0007	R^2 = -0.18*; *p* < 3.82e-16	R^2 = 0.14*; *p* < 3.82e-16		
Limbic	R^2 = 0.02; *p* = 0.12	R^2 = -0.15*; *p* <3.82e-16	R^2 = -0.15*; *p* <3.82e-16	R^2 = -0.24*; *p* < 3.82e-16	R^2 = 0.141*; *p* <3.82e-16	
Insular	R^2 = 0.04*; *p* = 2.89e-9	R^2 = 0.12; *p* = 0.52	R^2 = -0.10; *p* = 0.07	R^2 = -0.28*; *p* < 3.82e-16	R^2 = 0.09*; *p* = 7.38e-6	R^2= 0.06*; *p* = 0.003
