# Supplementary material for: Morphometric brain organization across the human lifespan reveals increased dispersion linked to cognitive performance
Source: PLoS Biol. 2024 Jun 20;22(6):e3002647. doi: 10.1371/journal.pbio.3002647 (PMC11189252; doi:10.1371/journal.pbio.3002647)
Supplement: S7 Table — All p values were corrected by FDR. (PDF) [file pbio.3002647.s016.pdf]

**Table S7. Age-related differences in between-network dispersion for four age windows, controlling for sex and eTIV. All *p* values were corrected by FDR.**

| | Adolescence | Young Adulthood | Middle Adulthood | Late Adulthood |
|---|---|---|---|---|
| Primary motor - Association1 | $t$ = 5.92*, $p$ = 4e-7 | $t$ = 0.86, $p$ = 0.48 | $t$ = -1.09, $p$ = 0.38 | $t$ = 2.72*, $p$ = 0.02 |
| Primary motor - Association2 | $t$ = 3.93*, $p$ = 0.0007 | $t$ = 0.64, $p$ = 0.61 | $t$ = -0.82, $p$ = 0.51 | $t$ = 0.43, $p$ = 0.75 |
| Primary motor - Secondary sensory | $t$ = 5.38*, $p$ = 3e-6 | $t$ = -2.67*, $p$ = 0.02 | $t$ = -3.74*, $p$ = 0.001 | $t$ = -1.23, $p$ = 0.33 |
| Primary motor - Primary sensory | $t$ = 3.81*, $p$ = 0.001 | $t$ = -2.62*, $p$ = 0.02 | $t$ = -3.68*, $p$ = 0.001 | $t$ = -1.20, $p$ = 0.33 |
| Primary motor - Limbic | $t$ = 2.91*, $p$ = 0.01 | $t$ = 0.53, $p$ = 0.69 | $t$ = 0.10, $p$ = 0.94 | $t$ = 1.31, $p$ = 0.32 |
| Primary motor - Insular | $t$ = -0.92, $p$ = 0.46 | $t$ = -1.11, $p$ = 0.38 | $t$ = 1.05, $p$ = 0.4 | $t$ = 2.78*, $p$ = 0.02 |
| Association1 -Association2 | $t$ = 0.25, $p$ = 0.88 | $t$ = -0.74, $p$ = 0.55 | $t$ = -1.23, $p$ = 0.33 | $t$ = 2.68*, $p$ = 0.02 |
| Association1 - Secondary sensory | $t$ = -1.38, $p$ = 0.29 | $t$ = -3.42*, $p$ = 0.003 | $t$ = -3.04*, $p$ = 0.009 | $t$ = -3.73*, $p$ = 0.001 |
| Association1 - Primary sensory | $t$ = -2.19, $p$ = 0.06 | $t$ = -3.19*, $p$ = 0.006 | $t$ = -0.48, $p$ = 0.72 | $t$ = 4.95*, $p$ = 2e-5 |
| Association1 - Limbic | $t$ = -4.38*, $p$ = 0.0001 | $t$ = -0.14, $p$ = 0.94 | $t$ = 0.31, $p$ = 0.84 | $t$ = -1.9, $p$ = 0.12 |
| Association1 - Insular | $t$ = -1.63, $p$ = 0.19 | $t$ = -0.90, $p$ = 0.46 | $t$ = -1.24, $p$ = 0.33 | $t$ = -1.28, $p$ = 0.32 |
| Association2 - Secondary sensory | $t$ = -3.66*, $p$ = 0.001 | $t$ = -3.22*, $p$ = 0.006 | $t$ = -2.59*, $p$ = 0.03 | $t$ = -0.10, $p$ = 0.94 |
| Association2 - Primary sensory | $t$ = -5.62*, $p$ = 1e-6 | $t$ = -2.8*, $p$ = 0.02 | $t$ = -1.39, $p$ = 0.29 | $t$ = 1.72, $p$ = 0.17 |
| Association2 - Limbic | $t$ = -5.17*, $p$ = 6e-6 | $t$ = -1.27, $p$ = 0.32 | $t$ = -0.92, $p$ = 0.46 | $t$ = 1.62, $p$ = 0.19 |
| Association2 - Insular | $t$ = -1.1, $p$ = 0.38 | $t$ = -1.36, $p$ = 0.29 | $t$ = -1.57, $p$ = 0.21 | $t$ = 0.09, $p$ = 0.94 |
| Secondary sensory - Primary sensory | $t$ = 2.40*, $p$ = 0.04 | $t$ = -0.03, $p$ = 0.98 | $t$ = 1.63, $p$ = 0.19 | $t$ = 4.71*, $p$ = 4e-5 |
| Secondary sensory - Limbic | $t$ = 0.21, $p$ = 0.89 | $t$ = -4.67*, $p$ = 4e-5 | $t$ = -3.06*, $p$ = 0.009 | $t$ = -0.91, $p$ = 0.46 |
| Secondary sensory - Insular | $t$ = 4.00*, $p$ = 0.006 | $t$ = -2.39*, $p$ = 0.04 | $t$ = -3.63*, $p$ = 0.002 | $t$ = -3.54*, $p$ = 0.002 |
| Primary sensory - Limbic | $t$ = 0.22, $p$ = 0.89 | $t$ = -2.01, $p$ = 0.10 | $t$ = 1.27, $p$ = 0.32 | $t$ = 5.33*, $p$ = 4e-6 |
| Primary sensory - | $t$ = 2.45*, | $t$ = -2.49*, | $t$ = -0.91, | $t$ = 2.71*, |

| | | | | |
|---|---|---|---|---|
| Insular | *p* = 0.04 | *p* = 0.03 | *p* = 0.46 | *p* = 0.02 |
| Limbic - Insular | *t* = 3.15*, *p* = 0.007 | *t* = 2.01, *p* = 0.1 | *t* = 0.53, *p* = 0.69 | *t* = -1.87, *p* = 0.12 |
